# Regulatory network reconstruction reveals genes with prognostic value for chronic lymphocytic leukemia

**DOI:** 10.1186/s12864-015-2189-6

**Published:** 2015-11-25

**Authors:** Sally Yepes, Maria Mercedes Torres, Liliana López-Kleine

**Affiliations:** Facultad de Ciencias, Departamento de Ciencias Biológicas, Universidad de los Andes, Bogotá D.C., Colombia; Departamento de Estadística, Universidad Nacional de Colombia, Bogotá D.C., Colombia

**Keywords:** CLL, IGVH mutational status, Regulatory networks

## Abstract

**Background:**

The clinical course of chronic lymphocytic leukemia (CLL) is highly variable; some patients follow an indolent course, but others progress to a more advanced stage. The mutational status of rearranged immunoglobulin heavy chain variable (IGVH) genes in CLL is a feature that is widely recognized for dividing patients into groups that are related to their prognoses. However, the regulatory programs associated with the IGVH statuses are poorly understood, and markers that can precisely predict survival outcomes have yet to be identified.

**Methods:**

In this study, (i) we reconstructed gene regulatory networks in CLL by applying an information-theoretic approach to the expression profiles of 5 cohorts. (ii) We applied master regulator analysis (MRA) to these networks to identify transcription factors (TFs) that regulate an IGVH mutational status signature. The IGVH mutational status signature was developed by searching for differentially expressed genes between the IGVH mutational statuses in numerous CLL cohorts. (iii) To evaluate the biological implication of the inferred regulators, prognostic values were determined using time to treatment (TTT) and overall survival (OS) in two different cohorts.

**Results:**

A robust IGVH expression signature was obtained, and various TFs emerged as regulators of the signature in most of the reconstructed networks. The TF targets expression profiles exhibited significant differences with respect to survival, which allowed the definition of a reduced profile with a high value for OS. TCF7 and its targets stood out for their roles in progression.

**Conclusion:**

TFs and their targets, which were obtained merely from inferred regulatory associations, have prognostic implications and reflect a regulatory context for prognosis.

**Electronic supplementary material:**

The online version of this article (doi:10.1186/s12864-015-2189-6) contains supplementary material, which is available to authorized users.

## Background

Chronic lymphocytic leukemia (CLL) is a heterogeneous disease with variable clinical manifestations and evolution [1]. Two major molecular subtypes are recognized, which are characterized by a high or low number of somatic hypermutations in the variable region of the immunoglobulin genes. This feature is known as the immunoglobulin heavy chain variable (IGVH) gene mutational status and is related to prognostic evolution, in which patients with an unmutated IGVH status have a less favorable prognosis than patients with a mutated IGHV gene [2, 3]. Other molecular biomarkers of progression in this disease include diverse cytogenetic rearrangements, gene mutations, and ZAP-70 expression [4–6]; however, these events do not appear to be fundamental agents in the leukemia process. Due to the importance of the IGVH status in disease course determination, several expression studies have focused on comparisons of the mutated IGVH vs. unmutated IGVH CLL forms [7–9]. However, these studies have identified genes that are not functionally related and therefore cannot elucidate biological mechanisms to distinguish between risk classes. Therefore, searching for the relevant prognostic biomarker surrogates for IGVH mutational status remains a necessity.

Several methods have been developed to identify expression signatures associated with prognosis. However, it is worth noting that markers are unstable and study dependent, often exhibiting poor overlap among studies and low classification power. According to Bae et al. [10], it is possible that expression signatures commonly contain cancer *drivers* and *passengers*, of which the latter are not directly involved in cancer progression. Therefore, it is of interest to search for regulators, such as transcription factors (TFs), that are causally responsible for the implementation of differential expression patterns and to evaluate their relation with progression and clinical outcome [11–13].

Here, in search of prognostic markers, we applied the ARACNE algorithm to find TFs that were involved in the differentiation process between IGVH subtypes. This algorithm is based on an information-theoretic approach that predicts potential functional associations among genes by identifying the statistical dependencies between their products [14]. ARACNE has been successfully applied in the search for master regulators and the study of clinical outcomes in different cancer models, the results of which can be subsequently validated through functional experiments. Some applications include the identification of c-MYC and BCL6 as critical genes in B-cell tumors [15, 16], master regulators with poor prognosis in breast cancer [11], cancer risk and master regulators for FGFR2 signaling [17], neuroblastoma and tumor progression [18] and multiple myeloma and its prognosis [19].

In this work, we reconstructed CLL regulatory networks using the ARACNE algorithm and used master regulator analysis (MRA) to identify candidate transcription factors that regulate an IGVH mutational status signature. The IGVH signature was developed through the identification of differentially expressed genes in a large number of samples using microarray meta-analysis. The principal intention here was to evaluate the implications of the inferred regulators and their targets for patient survival. Therefore, the candidate expression profiles were used to evaluate prognosis utilizing two measures of progression, time to treatment (TTT) and overall survival (OS).

The genes with prognostic implication identified in this work may represent reliable markers to predict outcomes because i) they were obtained through a method (MRA, which tested the significant intersections between the regulons that were represented in the ARACNE networks and the signature genes) that used as query a known prognostic marker in CLL, the IGVH mutational status, and ii) the genes that were inferred as key regulators exhibited significance using a Cox proportional hazards model with outcome indicators. Given the workflow, the identified genes reflect a regulatory context for prognosis rather than only differentially expressed genes.

## Methods

**Fig. 1:**
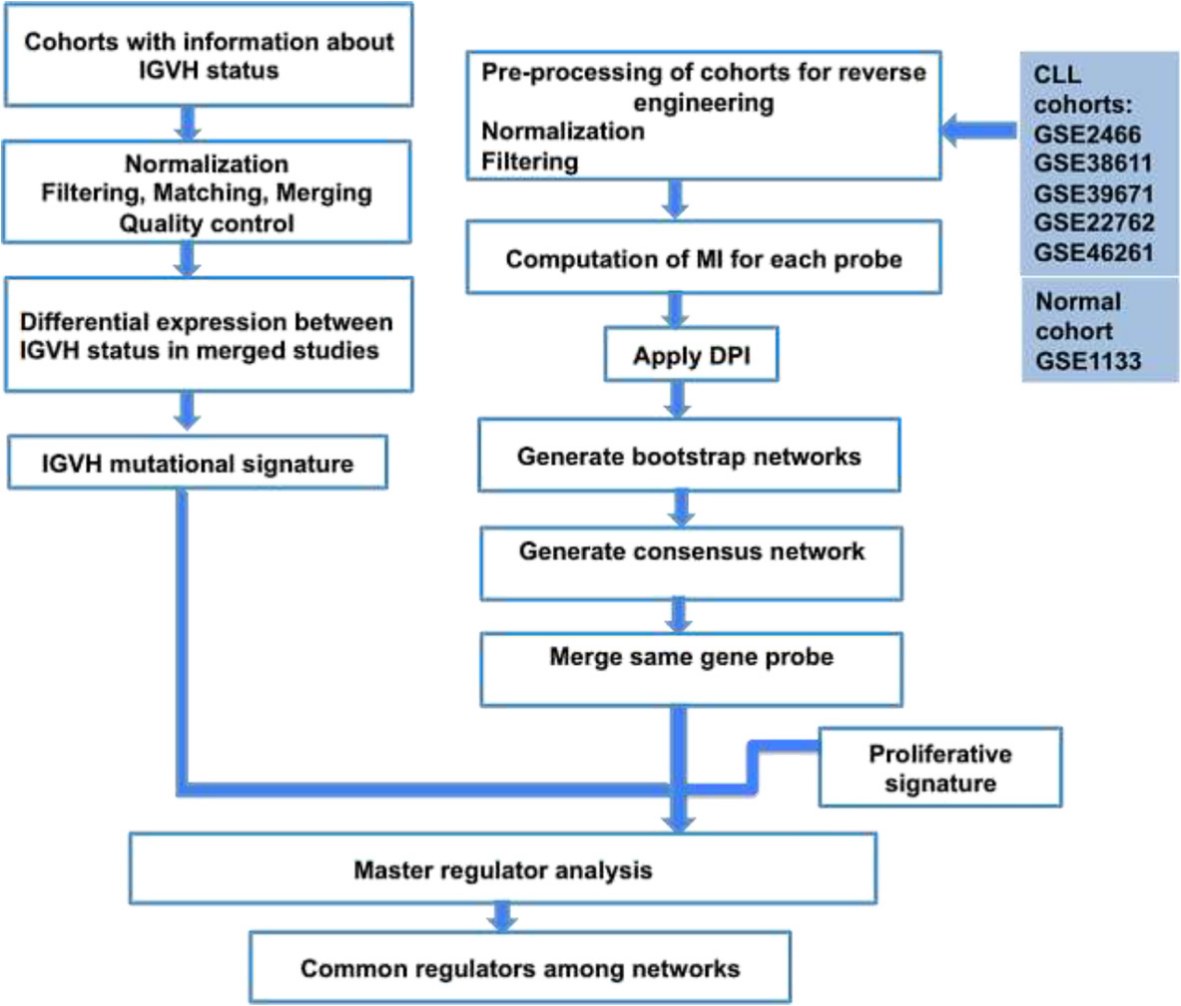
The workflow used for reconstructing the CLL regulatory networks and for regulator inference

A schematic description of the network reconstruction and regulator inference is represented in Fig. 1.

### Dataset

The present study used microarray gene expression data retrieved from the NCBI Gene Expression Omnibus (GEO) Database [20]. A total of 474 CLL patients from eleven studies were included in the differential expression analysis (Table 1). Each chosen dataset possessed available raw expression files (CEL files) and information regarding the subjects’ IGVH mutational status. Five cohorts with 100 or more CLL patients were used to reverse engineer the transcriptional networks.

**Table 1:** Cohorts used in this study

Author	Platform	Samples (mut/unmut)	Accession
Mosca L et al. [58]	HG_U133A	60 (23/37)	GSE16746
Fabris S et al. [59]	HG_U133A	60 (24/36)	GSE9992
Del Guidice et al. [60]	HG_U133A	20 (16/4)	GSE15777
Jao Baptista et al. [61]	HG_U133_Plus_2	24 (10/14)	GSE33135
Saiya-Cork K et al. [62]	HG_U133_Plus_2	19 (4/15)	GSE26526
Haslinger C et al. [63]	HG_U95A	100 (51/49)	GSE2466
Seifert M et al. [64]	HuGene 1.0 ST	9 (4/5)	GSE36907
	HG_U133_Plus_2	10 (5/5)	
Stamatopoulos B et al. [53]	HG_U133_Plus_2	14 (8/6)	GSE12734
Mukherjee P et al. [65]	HG_U133_Plus_2	22 (10/12)	GSE29605
Fabris S et al. [66]	HuGene 1.0 ST	136 (76/60)	GSE38611
Ronchetti D et al. [67]	HuGene 1.0 ST	211 (127/83)	GSE46261
Chuang HY et al. [68]	HG_U133_Plus_2	130 (NA)	GSE39671
Herold T et al. [49]	HG_U133_Plus_2	107 (NA)	GSE22762
Su AI et al. [40]	HG_U133A	126 (normal samples)	GSE1133

### Data preprocessing and IGVH mutational status signature

Probe level normalization was performed independently in each cohort using the VSN method [21]. Quality checks were performed before and after the normalization process. To obtain a robust result, we applied a gene filtering procedure to each study level, which removed 30 % of the non-expressed genes based on the mean intensity values and 30 % of the non-informative genes with a small variation based on variance.

Usually, different microarray platforms have multiple probes (or probe sets) that represent the same gene transcript; therefore, gene matching is necessary. For probe summarization, we used the “IQR” method, in which we selected the probe ID with the largest interquartile range (IQR) of the expression values to represent the gene. The number of genes in each study may be different; thus, we performed gene merging to extract the common genes across multiple cohorts. Additionally, we included some genes that appeared in 80 % of the studies and were missing in 20 % of the studies. The MetaDE package in R was used for filtering, matching and merging procedures [22].

To detect the differential expression between the IGVH subtypes, we used a microarray meta-analysis approach. This methodological framework increases the reliability and generalizability of results [23]. We used the “MetaOmics” software suite, which contains three unified R packages: MetaQC, MetaDE and MetaPath [22]. The MetaQC package [24] was used for determining the meta-analysis inclusion/exclusion criteria. MetaDE was used to apply various state-of-the-art genomic meta-analysis methods to detect differentially expressed (DE) genes, including the Fisher [25], Stouffer, adaptively weighted statistic (AW) [26], maximum *P*-value (maxP), and the rth ordered *P*-value (rOP) methods [27]. The R statistical environment [28] was used to perform all statistical analyses.

### Functional enrichment

The MetaPath package, which performs pathway meta-analysis [29], was used to detect enriched pathways. The Genecodis server [30–32] was used to perform modular enrichment analysis. The method obtains co-occurrence annotations in the KEGG and Panther databases, the *P* values are calculated through hypergeometric analysis corrected by FDR method.

### Reverse engineering of the transcriptional networks

After VSN normalization, each cohort was filtered based on its standard deviation distribution (sd). Probes with a sd below the shortest interval that contained half of the data in the distribution were discarded before the network reconstruction. ARACNE mutual information networks [14] were built based on five expression cohorts. Each network had 100 or more samples and was processed with different platforms (GSE2466, GSE38611, GSE39671, GSE22762, and GSE46261). ARACNE was used to infer the targets of 807 TFs that were represented in the gene expression profile. The algorithm uses information-theoretic methods to analyze physical transcriptional interactions between the TFs and their targets. ARACNE uses expression data to compute pairwise mutual information (MI) and employs a computationally efficient Gaussian kernel estimator. First, it eliminates interactions that are below a minimum MI threshold, and then the Data Processing Inequality (DPI) theorem is used to eliminate interactions that are considered sampling errors. R scripts from the original protocol calculated the kernel width and MI threshold parameters. The *P* value to determine the MI threshold and the DPI tolerance were set to 0.05 and 0 %, respectively. One hundred bootstrap datasets were used to create the bootstrap networks to accommodate the microarray data noise and the MI estimation error. A consensus network was then constructed by retaining edges that were supported across a significant number of the bootstrap networks. The entire process was also executed in normal tissues (GSE1133) as negative controls. The ARACNE algorithm was implemented in the Perl language by using the original procedure that was proposed by Margolin et al. [14], who also described the mathematical formulation of the algorithm.

### Regulator analysis

Master regulator analysis (MRA) [33] of the reconstructed networks was used to identify (TFs) that regulate an IGVH mutational status signature. Enrichment was evaluated using the Fisher’s exact test (FET); therefore, for each TF, the statistical significance of the intersection between the TF targets, which was represented in the ARACNE-generated network, and the list of differentially expressed genes was computed with the FET. These generated TFs were selected as candidate regulators of IGVH status. To avoid the possibility of finding non-specific CLL regulators and to exclude those that were directly involved with proliferation, we ran an MRA on both the CLL IGVH signature, to query a healthy tissue network, and a proliferative gene signature that was developed by Venet et al. [34], which is known as a meta-PCNA signature. To validate the power of the process for detecting significant regulons, we compared the common TFs among the reconstructed networks. MRA was performed using geWorkbench, a free, open-source genomic analysis platform [35].

### The clinical relevance and survival analyses

We applied the Global Test [36] to determine the association between expression profiles and survival. For cases in which a significant association with patient outcome was observed, the gene list was reduced to derive a smaller prognostic signature with the intention of providing a profile with potential clinical use. The smaller signature was constructed using Cox proportional hazards models and clustering analysis as proposed by Goeman JJ and Finos L [37]. Briefly, the methodology is based on regression models in which the distribution of the response variable (overall survival or time to treatment) is modeled as a function of the covariates (expression values) [36]. The covariates were ordered in a hierarchical cluster with the absolute correlation distance and the method average linkage. To reduce the profile or “zoom” in on the significant results, the process discards non-significant branches from the dendrogram with the corresponding covariates. The MLInterfaces package [38] was used to construct an SVM (support vector machine) [39] to assess the predictive power of the reduced profile. We used a 218-sample training set that was chosen at random from patients with good and poor prognoses and a 218-sample test set to calculate the classification error.

Pairwise t-tests were applied with Bonferroni *P* value corrections to compare the relative expression levels between two groups.

## Results

### The IGVH mutational status expression profile

Using a combination of CLL expression profiles, we used a microarray meta-analysis approach to obtain an IGVH mutational status signature. After preprocessing and quality control, we obtained 12,487 genes in 436 CLL patients from eight different cohorts, which included 218 unmuted and 218 mutated IGVH samples.

GSE26525 and GSE36907 were determined to be of lower quality after six quantitative quality control (QC) measures were taken into consideration; therefore, they were removed from the meta-analysis. The QC measures utilized were proposed by Kang et al. [24] and included: covering the internal homogeneity of coexpression structures among studies, the external consistency of coexpression patterns within a pathway database, and the accuracy and consistency of expressed gene detection or enriched pathway identification. Each QC index was used to identify low-quality studies and to determine whether they should be excluded from the meta-analysis.

**Fig. 2:**
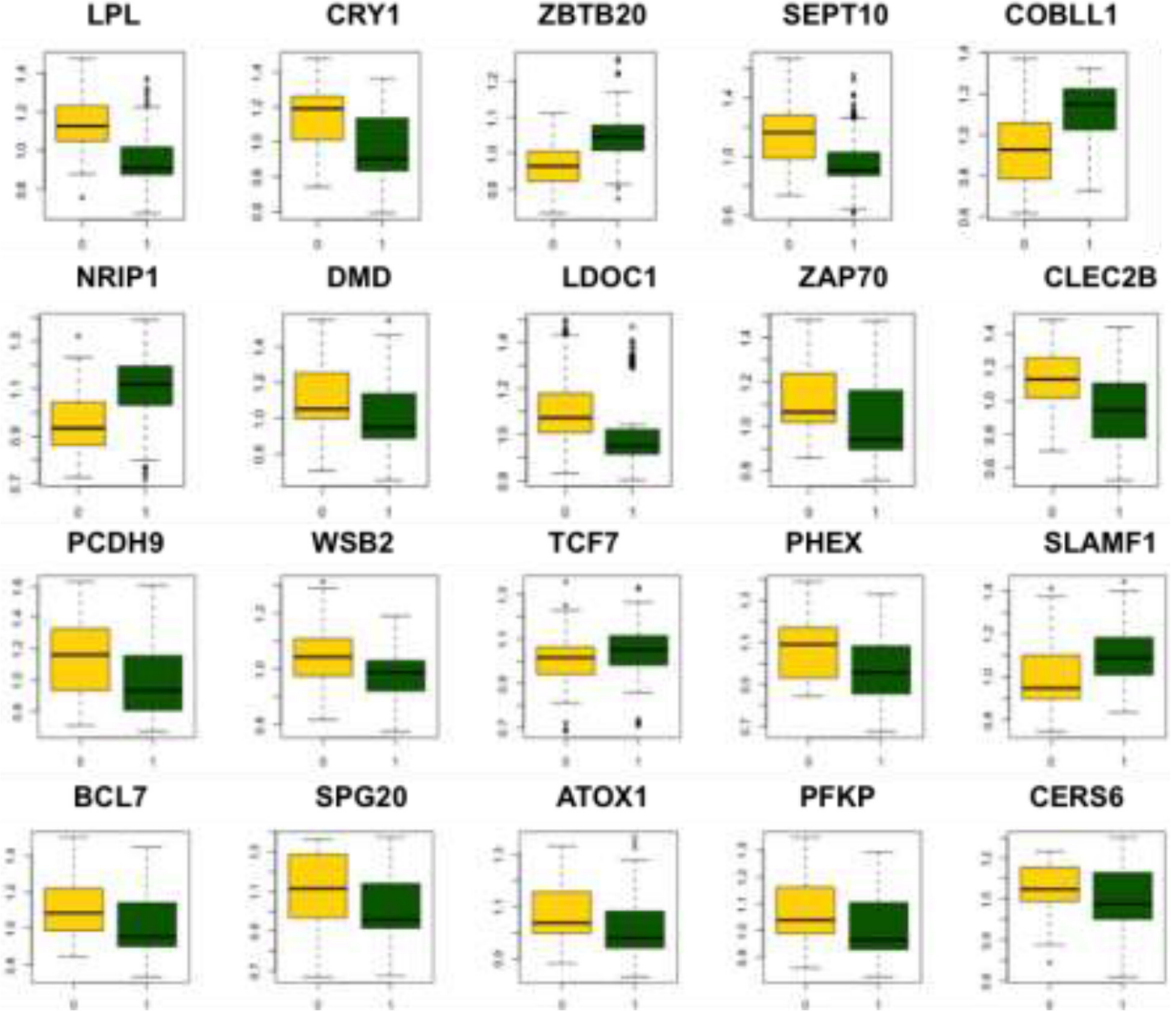
Expression distribution for the mutated and unmutated IGHV samples with respect to the top 20 differentially expressed genes (total samples: 436). The y-axis represents relative expression (normalized sample values divided by the mean of each gene across all samples). The x-axis represents the IGVH status of the unmutated (yellow) and muted (green) patient groups. The boxplot bars indicate the lower and upper quartiles, the central bars indicate the mean, the whiskers indicate one standard deviation of the mean, and the box widths are proportional to the sample size. All genes were observed to be significantly different (*p* > 0.001)

The Fisher *P*-value method detected a significant number of genes and was used in the meta-analysis for differential expression. Table 2 shows the top 20 genes with differential expression between the IGVH statuses as determined using the Fisher method, and these are listed in order of statistical test and significance level. The total list of genes that were determined with statistically significant differences can be found in Additional file 1: Table S1. Figure 2 shows the expression distribution of the first 20 differentially expressed genes that were found with the meta-analysis with respect to IGVH status. Significant differences between the sample groups were observed for all the top genes (*P* > 0.001, two-tailed pairwise *t*-test with Bonferroni corrections).

**Table 2:** The top differentially expressed genes obtained with the meta-analysis

Gene	Regulation in poor prognoses	Corrected *P*-value
CRY1	Up	4.78e-19
LPL	Up	4.78e-19
ZBTB20	Down	4.78e-19
SEPT10	Up	4.78e-19
COBLL1	Down	4.78e-19
NRIP1	Down	4.78e-19
DMD	Up	4.78e-19
ZAP70	Up	4.78e-19
LDOC1	Up	4.78e-19
WSB2	Up	4.78e-19
CLEC2B	Up	4.78e-19
PCDH9	Up	4.78e-19
TCF7	Down	4.78e-19
PHEX	Up	4.78e-19
SLAMF1	Down	4.78e-19
BCL7A	Up	4.78e-19
PFKP	Up	4.78e-19
ATOX1	Up	4.78e-19
USP6NL	Down	4.78e-19
FUT8	Down	4.78e-19
SPG20	Up	4.78e-19
TGFBR3	Up	4.78e-19
CERS6	Up	4.78e-19
FLNB	Up	4.78e-19
P2RX1	Up	4.78e-19
MYBL1	Down	4.78e-19
RNF41	Up	4.78e-19
IFI44	Up	4.78e-19
FADS3	Up	4.78e-19

Using a modular enrichment analysis with the KEGG and Panther pathways and the list of differentially expressed genes (*p* < 0.05), the following co-occurrence annotations were found with low significant *P* values: pathways in cancer, focal adhesion, T cell receptor signaling, angiogenesis, and MAPK signaling. Pathway detection using the MetaPath package and collections from the molecular signatures database (MSigDB) showed a significant result for the GO terms that were related to extracellular signaling (q-value >0.2). The following terms were found to be statically significant: proteinaceous extracellular matrix, extracellular region, extracellular space, extracellular matrix, and extrinsic to membrane.

### Reconstruction of the CLL transcriptional networks and regulator analysis

In Table 3, we list the datasets that were used for the regulatory network reconstructions, including the number of probes after filtering, which ensured the use of highly variable probes, and the large number of interactions (edges) and targets (nodes) found in each network after the reconstruction.

**Table 3:** Metrics of the reconstructed networks

Dataset accession	No. of samples	Array platform	No. probes (original array /after filtering)	Nodes	Edges
GSE2466	100	HG_U95A	12,626/8,879	6,494	27,207
GSE38611	136	HuGene 1.0 ST	32,321/22,454	17,271	110,952
GSE39671	130	HG_U133_Plus_2	54,675/39,255	12,115	78,351
GSE22762	107	HG_U133_Plus_2	54,675/38,868	16,471	169,016
GSE46261	211	HuGene 1.0 ST	32,321/12,968	12,289	57,587
GSE1133	126	HG_U133A	22,283/13,941	9,428	51,609

To improve the specificity of the regulator analysis, we conducted control processes. We used the IGVH status signature to query a healthy tissue network (GSE1133) [40] and used it as a control to detect regulators that were not tissue-specific. In addition, to exclude the regulators that were involved with proliferation, we performed the MRA within the CLL networks using as query a PCNA proliferative gene signature [34]. No inferred TF was enriched in the healthy control networks, nor were any TFs involved with the proliferative signature that was tested. Therefore, the specificity checks confirmed that the TFs involved in the prediction were related to CLL pathology.

The MRA identified a relatively small number of regulators in each network, and some variation was observed among them (Table 4). In total, 35 TFs were identified after taking into account all networks. With respect to the pathways enriched within this group of TFs, a co-occurrence annotation was found for Wnt signaling (0.00018). In spite of this variation, the following TFs emerged as regulators in at least four networks: CERS6 (80 targets), TCF7 (95 targets), and MYBL1 (59 targets). The number shown in parenthesis includes the targets in total for all the networks. Overlap among these selected regulators and their targets was found, in which 20 to 28 target genes were shared; consequently, multiple genes in the IGVH signature were co-regulated by several TFs (Additional file 1: Table S2).

**Table 4:** Transcription factors in CLL regulatory networks after MRA

Network GSE46261				Network GSE39671			
Master regulator	FET *P*-value	Inter-section set	Mode	Master regulator	FET *P*-value	Inter-section set	Mode
CERS6	9.01E-36	40	+	EGR3	7.36E-17	39	-
TCF7	1.17E-28	37	-	CERS6	8.80E-17	47	+
TLE1	1.66E-16	22	+	PHTF1	1.22E-11	37	+
ZNF135	3.97E-15	21	+	AEBP1	4.51E-10	30	+
MYBL1	4.33E-13	22	-	NR2F6	4.58E-08	20	+
ELK3	3.59E-10	16	+	MYBL1	7.17E-08	26	-
AEBP1	1.11E-09	16	+	ZNF91	8.43E-08	16	-
GFI1	3.00E-09	13	+	TFDP1	3.64E-07	16	+
NCOR2	4.86E-08	12	+	TCF7	5.57E-07	42	-
TRPS1	6.00E-08	11	+	APEX1	2.00E-06	24	+
TSHZ2	3.55E-07	16	-	ZNF354A	3.26E-06	10	+
ARID5B	6.01E-07	9	-	SMARCA4	6.54E-06	39	+
NOD2	7.06E-07	17	+	Network GSE38611			
EGR3	1.20E-06	9	-	MYBL1	3.80E-07	6	-
ZNF236	2.13E-06	8	+	CERS6	5.90E-06	4	+
HOXB2	2.76E-06	9	+	Network GSE22762			
BRIP1	3.27E-06	15	-	TCF7	3.01E-23	49	-
MTA1	6.51E-06	14	+	EGR3	5.25E-14	23	-
Network GSE2466				CERS6	1.21E-11	21	+
KLF7	2.11E-13	23	-	MYBL1	3.38E-10	24	-
AFF1	1.79E-08	16	-	TCF7L2	3.82E-10	31	-
TCF3	1.26E-07	21	+	MYBL2	8.67E-10	23	+
TCF7	1.77E-05	18	-	AEBP1	1.89E-09	16	+
TLE1	2.72E-05	8	+	TFEC	6.31E-08	12	-
RUNX3	5.22E-05	13	-	PHTF1	8.18E-08	29	+
ZNF135	1.10E-04	8	+	PBX3	7.17E-07	14	+
KLF10	1.85E-04	6	+	EP400	7.72E-07	17	+
CERS6	1.85E-04	6	+	PPARD	5.82E-06	25	-

### The clinical relevance of regulators with respect to survival

Expression profiles were tested to evaluate their relationship with outcome using the GSE22762 (*n* = 107) and GSE39671 (*n* = 130) datasets. As seen in Table 4, some TF targets were found in common in the reconstructed networks. CERS6, TCF7 and MYBL1 stood out, suggesting a unifying process in CLL progression. Therefore, we focused on these regulons for interpretation. Every profile (TF targets) independently tested exhibited significance for survival with the following respective *P* values for OS and TTT: TCF7 (*P* = 4.21e-08, *P* = 0.0046), CERS6 (*P* = 3.13e-06, *P* = 0.015) and MYBL1 (*P* = 5.04e-5; *P* = 0.002).

Then, we tested whether CERS6, TCF7, MYBL1 and their targets as a group, which included a total of 166 genes, could be related to patient outcomes. We found a significant difference for OS (*P* = 2.46e-07) and for TTT (*P* = 0.00548). In Table 5, genes associated with OS survival are listed in order of significance, as well as the direction of their regulation, and genes with statistical significance in both cohorts are underlined. TCF7 had prognostic value in both cohorts, and it was the second-most statistically significant gene in the GSE22762 cohort. Additionally, NRIP1 and PDE8A were at the top of the list. All three genes were downregulated with respect to poor prognosis. Enrichment analysis using the 166 genes showed that focal adhesion (2.3e-08) and T cell receptor signaling (2.6e-06) were the most implicated pathways, and the MAPK and Wnt signaling pathways, among others, were also detected with significant corrected *P* values.

**Table 5:** Transcription factors and targets with significant overall survival associations in cohort GSE22762

GENE	Regulation in poor prognoses	*P*-value	GENE	Regulation in poor prognoses	*P*-value
NRIP1	Down	1.89E-10	BCL7A	Up	9.31E-04
TCF7	Down	1.67E-06	SNED1	Down	1.09E-03
PDE8A	Down	7.10E-06	DOK2	Down	1.10E-03
CD247	Down	7.67E-06	ARSD	Up	1.14E-03
SORL1	Down	4.27E-05	RASGRP1	Down	1.27E-03
ATOX1	Up	5.07E-05	LHFPL2	Down	1.54E-03
P2RX1	Up	6.37E-05	EGR3	Down	1.65E-03
NME1	Up	8.27E-05	HLADMA	Up	1.85E-03
NMB	Up	1.12E-04	LRMP	Up	1.96E-03
GMDS	Up	1.17E-04	DIP2C	Up	2.06E-03
IL2RB	Down	1.34E-04	TMED3	Up	3.24E-03
AAK1	Down	1.61E-04	MYBL1	Down	3.29E-03
ME2	Up	1.76E-04	PHEX	Up	3.81E-03
SERPINF1	Up	3.00E-04	UGT8	Up	4.98E-03
FARP1	Up	3.27E-04	SDC3	Down	5.25E-03
NUCB2	Down	3.58E-04	SFTPB	Up	6.06E-03
HOMER2	Up	5.74E-04	PEBP1	Up	6.34E-03
SLC16A6	Down	5.75E-04	LPL	Up	6.98E-03
SYNJ2	Down	9.05E-04	LDOC1	Up	7.04E-03
SLAMF1	Down	9.20E-04			

Genes with significant time to treatment associations in cohort GSE39671 are underlined

**Fig. 3:**
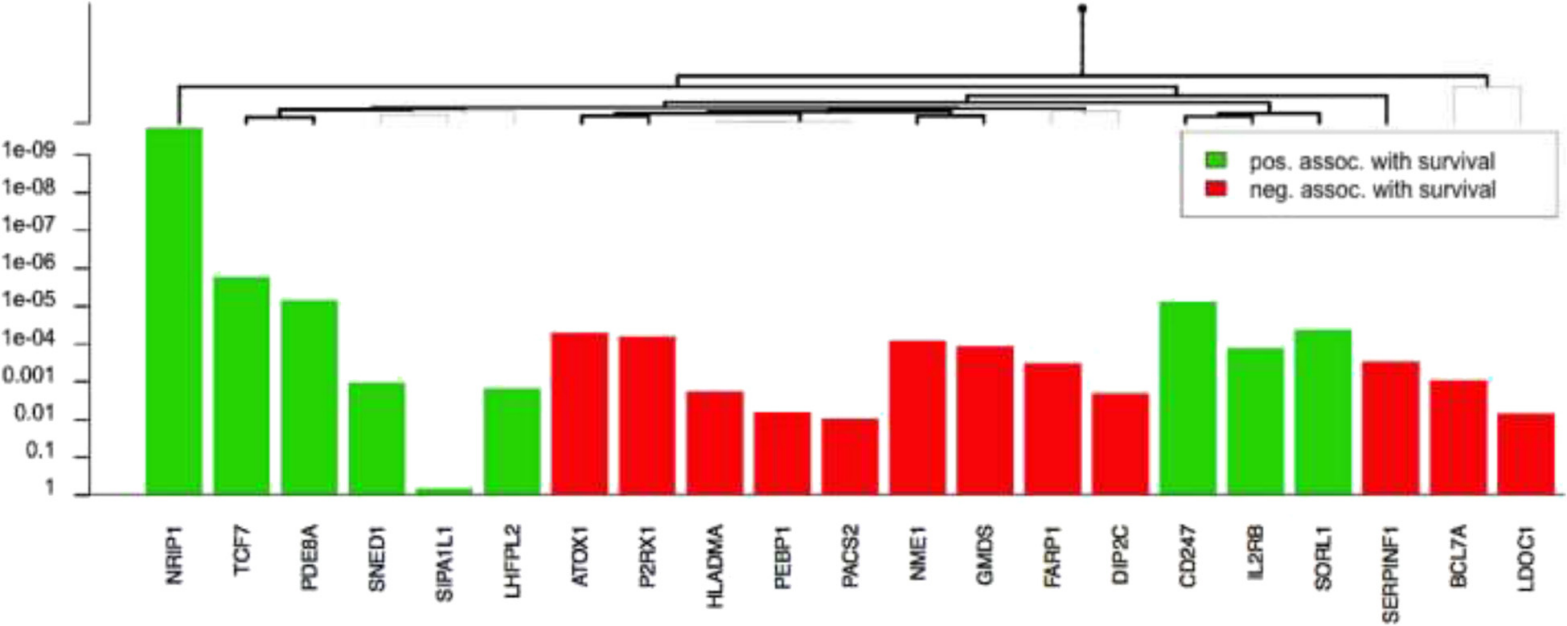
Reduced profile with a high value for OS. The profile was obtained using proportional hazards models constructed for 3 regulators: TCF7, CERS6, MYBL1 and their targets. The direction [green: positive (POS); red: negative (NEG)] and survival correlation significance (P values on the y-axis) are indicated

We next reduced the expression profile to develop a smaller prognostic gene signature using a Cox proportional hazards model. From the 166 genes, the procedure reduced the profile to 20 genes with positive or negative associations with survival. As shown in Fig. 3, the genes are ordered in a hierarchical clustering graph, which only shows the significant branches of the reduced profile. Notably, NRIP1 had the highest statistical significance, followed by TCF7, and the high expression of both genes was associated with survival. In other words, low expression was associated with a poor prognosis.

**Fig. 4:**
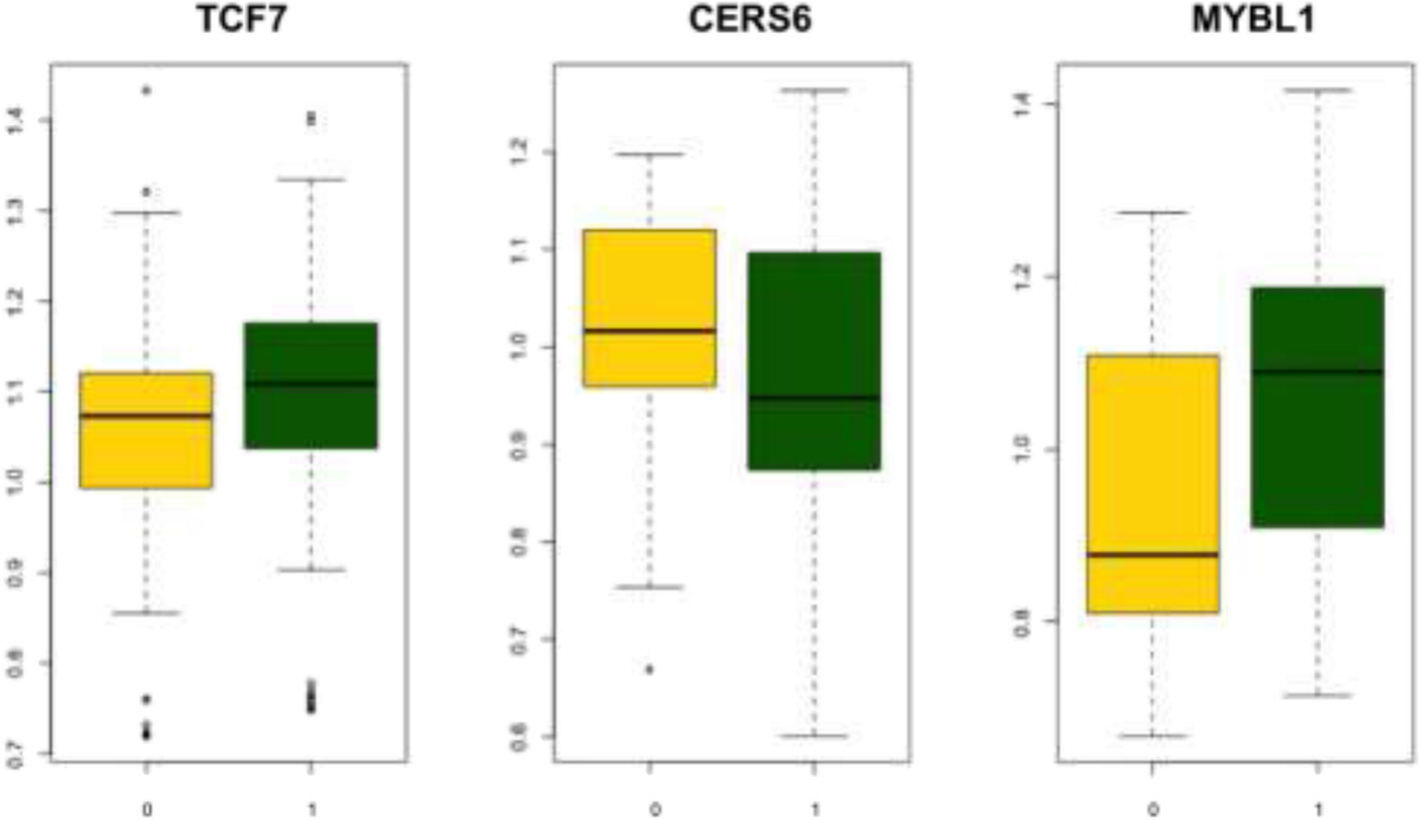
TCF7, CERS6, and MYBL1 expression levels from the integrated dataset (total samples: 436). The y-axis represents relative expression (normalized sample values divided by the mean of each gene across all samples). The x-axis represents the unmutated (yellow) and muted (green) IGVH statuses. The boxplot bars indicate the lower and upper quartiles, the central bars indicate the mean, the whiskers indicate one standard deviation from the mean, and the box widths are proportional to the sample sizes. All genes were significantly different (*p* > 0.001, two-tailed pairwise t-tests with Bonferroni corrections) between the sample groups

To determine whether the CERS6, TCF7 and MYBL1 expression levels were related to IGVH mutational status, we used pairwise t-tests to analyze the complete dataset that was previously used for the meta-analysis. We found that TCF7 and MYBL1 expression was significantly lower in the unmutated IGVH status patients than in the mutated IGVH status patients. Moreover, CERS6 expression was higher in the unmutated patients, indicating that these genes play an important role in disease prognosis (Fig. 4).

An SVM classifier was constructed for the common regulators (TCF7, CERS6, and MYBL1) and their targets, and the top 50 IGVH signature DE genes. We compared the dataset previously used in the meta-analysis (436 samples) with the classification error rates from the confusion matrix. The regulators and the 50-gene signature performed similarly, although a slightly lower error was observed in the signature (0.09174312 vs. 0.05504587). These classification errors indicate that the regulators and their targets exhibited prognostic utility.

## Discussion

To identify prognostic markers in CLL, a robust IGVH mutational signature was generated and used to infer its upstream TFs through mutual information networks and master regulator analysis. Given the large number of samples used and the heterogeneous spectrum of genomic aberrations represented, the integrated information allowed us to bring together various molecular events that underlie CLL and to compare patients only by their IGVH mutational status.

Because the IGVH mutational status is a good survival predictor, genes that are differentially expressed in mutated versus unmutated subtypes are also meaningful in prognosis. The top differentially expressed genes found in this study have been previously associated with IGVH status [7–9]. Genes such as LPL, CRY1, ZBTB20, SEPT10, COBLL1, NRIP1, DMD, LDOC1, and ZAP70 were found in the top DE list and have been shown to have CLL prognostic value [41, 42].

The differentially expressed genes between the IGVH subtypes were preferentially enriched with the pathways that are related to extracellular signaling. As was shown in the pathway meta-analysis, no specific differences between the subtypes at the specific pathway level were observed; therefore, it is possible that a significant overlap in the molecular characteristics in the IGVH subtypes were present. Previous expression profiling that was conducted with small patient numbers showed a common gene expression signature and reduced number of differentially expressed genes between IGVH subtypes [7, 8]. Although the number of differentially expressed genes increased in our meta-analysis approach, no difference was observed in the particular pathways beyond the terms related with extracellular signaling. These findings suggest that both subtypes of the disease are derived from a common origin or common transformation mechanism. It has previously been suggested that the IGVH subtypes derive from progenitors that are reminiscent of antigen-experienced B cells given the similar expression profiles [7], a model supported by frequent B cell receptor repertoire skewing and stereotypy [43].

The modular enrichment analysis executed directly with all the differentially expressed genes, improved the resolution of the pathway analysis, co-occurrence annotations were found with low significant *P* values for: pathways in cancer, focal adhesion, T cell receptor signaling, angiogenesis, and MAPK signaling. The pathways involved suggest the cellular origin of CLL. Encounter of naive B cells with antigen may progress either through a T cell-dependent reaction or in T cell-independent immune response. Possibly the mutated IGVH subtype is derived from the post-germinal center, generating memory B cells that have undergone somatic hypermutation of IGHV genes, unlike the unmutated subtype, which has not passed through the germinal center reaction, leading to the formation of antigen-experienced B cells harboring unmutated IGHV genes [2]. The expansion of CLL cells may be due to the accumulation of genetic lesions that confer higher aggressiveness, as well as interactions with the micro-environmental and antigens through the BCR, that promote signaling associated with cell proliferation and apoptosis inhibition [43].

Some differences in the chromosomal rearrangements between the subtypes, such as 17q and 11q deletions, as well as mutations in the ATM, are thought to be associated with the unmutated subtype, while less severe changes, such as 13q deletions, are associated with the mutated IGVH subtype. Additionally, the use of certain VH genes suggests differences in antigenic stimulation (i.e., VH 1–69 in the non-mutated subtype vs. VH 3–7, 4–34 in the mutated subtype) [44]. Nonetheless, it is not clear if the differences mentioned above are caused by the mutational IGVH status or are associated with it.

CERS6, TCF7, and MYBL1 stood out as common regulators in at least four networks, all three TFs have been implicated in the cancer process. CERS6 is involved in pro-apoptotic responses [45], epithelial-to-mesenchymal transition, plasma membrane fluidity and cell motility [46].

TCF7 is a member of a family of HMG-box-containing factors that are known to associate with β-catenin in the nucleus to mediate Wnt signaling. The Wnt signaling pathway is activated in CLL, and our data strengthens its role in prognosis. Uncontrolled Wnt signaling may contribute to the defective apoptosis that characterizes this malignancy [47]. Recent evidence regarding the role of TCF7 in CLL has been reported; in multivariate analyses of CLL patients, Kienle et al. [48] found evidence for the role of TCF7 in genetic risk defined by IGHV status, V3-21 usage, 11q-, 17p- and survival. Herold et al. [49] proposed an eight-gene prognostic score for CLL that included TCF7, NRIP1, and PDE8A for the prediction of survival and TTT. Here, we observed that these three genes were highly associated with survival using a completely different methodological approach and the same cohort (GSE22762). Conversely, Bou et al. [50] proposed a risk score combining with NRIP1 and TCF7 expression to identify high-risk patients. Therefore, the involvement of these genes in disease prognosis is significant.

The TCF7 targets inferred in this work were compared with the results of Wu et al. [51], who found that this TF was implicated in self-renewal and differentiation switch in early hematopoietic precursors. The authors used ChIP-Seq analysis to identify target genes bound by TCF7 in a multipotential hematopoietic cell line. Thirty-nine inferred targets in our work were identified by ChIP-Seq analysis in the above-mentioned work. Of these, the following are part of the reduced profile: BCL7A, CD247, GMDS, LHFPL2, NME1, NRIP1, and TCF7. To some extent, these results validate the inferred interactions.

Another regulator that was consistently found in several networks was MYBL1. This is a TF that plays a role in B-cell hematological malignancies [52]. Stamatopoulos et al. [53] found that MYBL1 expression predicted overall survival in CLL patients in the context of ZAP70 expression. As evident in the IGVH signature developed here (Table 2), MYBL1 was under-expressed in the unmutated subtype, emerged as a key regulator in the process of regulator inference (Table 4), and was associated with poor prognosis (Table 5). Interestingly, MYBL1 is specifically expressed by centroblasts [54]; therefore, this gene may be involved in the centro-germinal reaction and may support the cellular origin of CLL. On the other hand, common mechanisms should operate in CLL and diffuse large B-cell lymphoma (DLBCL) because, for example, some cases of DLBCL occur in a CLL background (Richter’s Syndrome). Interestingly, MYBL1 is part of a gene expression-based risk score in DLBCL [55], and it is included in outstanding molecular signatures developed for molecular subclassification in DLBCL [56, 57].

It is recognized that the overlap between the expression studies, particularly with respect to the prognostic signatures, is not perfect and contains only a few genes. Prognostic signatures have proven to be study dependent and inconsistent. Lim WK et al. [11] argued that genes in prognostic signatures act as *passengers* rather than *drivers* of the phenotypic differences. The genes that are most differentially expressed among the phenotypic states tend to be downstream from the determinants of the differences. Due to the complex interplay of regulatory interactions, these downstream genes are unstable. Therefore, it is of interest to search for transcription factors that regulate prognostic signatures.

## Conclusions

Regulatory network reconstructions allowed us to identify candidate regulators for an IGVH signature and uncover markers with prognostic implications. Moreover, with the nature of the methodological process, our results provide some insight into the regulatory programs that are involved.

## Additional file

Additional file 1: Table S1. Total list of genes with statistically significant differences obtained from the meta-analysis. **Table S2.** Selected regulators (CERS6, TCF7, and MYBL1) and their targets. (XLSX 132 kb)

## Abbreviations

CLL: Chronic lymphocytic leukemia
IGHV: Immunoglobulin variable region heavy chain
DE: Differentially expressed
TF: Transcription factor
MRA: Master regulator analysis
SVM: Support vector machine
OS: Overall survival
TTT: Time to treatment
